# Evolutionary morphology in shape and size of haptoral anchors in 14 *Ligophorus* spp. (Monogenea: Dactylogyridae)

**DOI:** 10.1371/journal.pone.0178367

**Published:** 2017-05-24

**Authors:** Abril Rodríguez-González, Volodimir Sarabeev, Juan Antonio Balbuena

**Affiliations:** 1 Marine Zoology Unit, Cavanilles Institute of Biodiversity and Evolutionary Biology, Science Park, University of Valencia, Paterna, Valencia, Spain; 2 Department of Biology, Zaporizhzhia National University, Zhaporizhzhia, Ukraine; Charles University, CZECH REPUBLIC

## Abstract

The search for phylogenetic signal in morphological traits using geometric morphometrics represents a powerful approach to estimate the relative weights of convergence and shared evolutionary history in shaping organismal form. We assessed phylogenetic signal in the form of ventral and dorsal haptoral anchors of 14 species of *Ligophorus* occurring on grey mullets (Osteichthyes: Mugilidae) from the Mediterranean, the Black Sea and the Sea of Azov. The phylogenetic relationships among these species were mapped onto the morphospaces of shape and size of dorsal and ventral anchors and two different tests were applied to establish whether the spatial positions in the morphospace were dictated by chance. Overall significant phylogenetic signal was found in the data. Allometric effects on anchor shape were moderate or non-significant in the case of evolutionary allometry. Relatively phylogenetically distant species occurring on the same host differed markedly in anchor morphology indicating little influence of host species on anchor form. Our results suggest that common descent and shared evolutionary history play a major role in determining the shape and, to a lesser degree in the size of haptoral anchors in *Ligophorus* spp. The present approach allowed tracing paths of morphological evolution in anchor shape. Species with narrow anchors and long shafts were associated predominately with *Liza saliens*. This morphology was considered to be ancestral relative to anchors of species occurring on *Liza haematocheila* and *M*. *cephalus* possessing shorter shafts and longer roots. Evidence for phylogenetic signal was more compelling for the ventral anchors, than for the dorsal ones, which could reflect different functional roles in attachment to the gills. Although phylogeny and homoplasy may act differently in other monogeneans, the present study delivers a common framework to address effectively the relationships among morphology, phylogeny and other traits, such as host specificity or niche occupancy.

## Introduction

Darwin’s view of species as evolving entities only detectable by gaps in morphological variation [1] established an explicit link between morphology and evolution. This inception has pervaded biological thought until today, to the point that it can be asserted that all post-Darwinian morphology has been, to a greater or lesser extent, evolutionary [2]. In comparative morphology, the relationship between morphology and evolution is assessed by identifying homologies and determining the chronological order of transformations of evolutionary units [2]. The similarity among forms of different species can be explained by inheritance from a common ancestor or by convergence where the form can arise more than once across taxa in response to similar ecological, adaptive, functional, and/or developmental pressures [3, 4]. Both processes act concurrently and disentangling their roles has been until recently a daunting task. However, the current availability of phylogenetic tools, coupled with the development of geometric morphometrics methods that can examine morphological data as independent from the effect of phylogeny have greatly simplified this endeavour [5, 6].

Historically, the tendency for related species to resemble one another more than species drawn at random from the same tree has been termed “phylogenetic signal” [5, 7]. Hence, determining the degree to which traits exhibit phylogenetic signal is crucial to understand how species vary phenotypically and to infer the evolutionary processes that have shaped their phenotypic diversity over evolutionary time [8]. In addition, to allow controlling for the confounding effect of phylogenetic dependence, estimation of phylogenetic signal provides a predictive framework of the value of a given trait for a species or an ensemble of closely related species based on their phylogenetic position [5]. The latter is important for parasites because their small size and cryptic natural history hampers the estimation of phenotypic and ecological traits [9]. However, phylogenetic signal in parasites has rarely been the focus of rigorous analyses [10–12]. Most studies have been chiefly based on comparison of ecological traits, such as abundance and host specificity to investigate diversification, diversity and community ecology [9, 10, 12–16], whereas few have considered morphological traits [17, 18].

Haptoral structures in Monogenea provide an exceptional platform for comparative morphology. On the one hand, and as in any other set of organisms, phylogenetic constraints are expected to account for morphological similarity between species. In fact, haptoral morphology has been found to be suitable for inferring phylogenetic relationships in different monogenean taxa [17, 19, 20–22]. On the other hand, the attachment structures of monogeneans are subjected to strong selective pressures. In gill monogeneans, these pressures are exerted by both the structural complexity of fish gills, thereby offering a wide variety of microhabitats, and exposure to mechanical stress generated by ventilating currents [23]. In fact, Šimková et al. [24] posit that the morphology of the haptor is, to a large degree, determined by adaptation to the host (host specificity) and to specific sites within their hosts (niche preference), which has been corroborated in, for instance, *Lamellodiscus* spp. [25]. However, other studies indicate that haptor morphology seems to be driven by a combination of both adaptive forces and phylogenetic constraints [26]. For instance, Rodríguez-González et al. [27] showed that different modular arrangements in the anchors of *Ligophorus* spp. could be accounted for by both adaptive and phylogenetic factors acting at different levels.

*Ligophorus* represents a genus of gill monogeneans exclusive to grey mullets (Osteichthyes: Mugilidae). This host-parasite system has several features that make it invaluable as a model system for studying the evolutionary processes that drive its past diversification and present diversity [18, 22]. The genus is speciose (59 valid species) and morphologically diverse [28, 29]. Well-resolved phylogenies are available [18, 22, 30] and specimens can be easily obtained in large numbers. *Ligophorus* spp. exhibit strict host specificity and several congeneric species tend to occur on the same hosts [22, 29, 30]. Geometric morphometrics has already been applied to *Ligophorus* spp. to explore the correlation between phenotypic variation in attachment organs and factors such as phylogeny, to elucidate mechanisms determining phenotypic buffering, character displacement, as well as in species discrimination [18, 29–32].

In the present paper, we evaluate the relationship between the form (i.e., the combination of shape and size) [33] of haptoral anchors and phylogeny of 14 species of *Ligophorus* from the Mediterranean Sea, Black Sea and Sea of Azov. This question has already been addressed by Khang et al. [18] in 13 *Ligophorus* spp. from Malaysia, where strong correlation between anchor shape variation and phylogeny was found. However, their study was geographically constrained to the Malay Peninsula and involved two host species only. In fact, their *Ligophorus* spp. were distributed in two clades corresponding to host species and, therefore, it is difficult to determine whether, and to which extent, morphological differences between the two clades reflect phylogeny or adaptation to host species.

Our study model is more complex, involving six host species and several host-switches [22, 30], allowing testing more elaborate hypotheses. For instance, if adaptation to branchial morphology of the host species were a decisive driver of haptoral morphology, it would be expected that anchor form of the switched species differs substantially from that of their closest phylogenetic relatives and be similar to that of other species occurring on the same host species. Alternatively, if phylogeny were the major determinant, anchor morphology would remain relatively constant within the clade and will differ from that of more distant species co-occurring on the same host.

In this study we specifically use tools of geometric morphometrics that can be applied in the phylomorphospace and multivariate statistical tests with the aim of quantifying phylogenetic signal in shape and size in ventral and dorsal anchors in 14 species of *Ligophorus* in order to determine the relative weights of convergence and shared evolutionary history, driving anchor form within the genus. We illustrate how the search for phylogenetic signal in morphological traits combined with multivariate statistics can improve our understanding of evolutionary morphology in Monogenea and parasites in general.

## Materials and methods

### Ethics statement

The fishes needed for the study were obtained within day-to-day fishery operations and purchased dead from licensed commercial fishermen or local fish markets. The number of fish host used (77) was kept to a reasonable minimum to guarantee the success of the research (see S1 Table). Grey mullets are locally and globally abundant and are not subjected to special conservation regulations in Spain, Russia and Ukraine, and the species involved—*Mugil cephalus* L., 1758, flathead grey mullet, *Liza saliens* (Risso, 1810), leaping mullet, *Liza ramada* (Risso, 1827), thinlip grey mullet, *Liza aurata* (Risso, 1810), golden grey mullet, *Chelon labrosus* (Risso, 1827), thicklip grey mullet, and *Liza haematocheila* (Temminck and Schlegel, 1845), so-iuy mullet—are listed by the IUCN as “Least Concern”.

### Sample composition

We based our morphological analysis on 286 individuals belonging to 14 of 16 valid species of *Ligophorus* (about 23% of all known species of the genus) recorded in the Mediterranean, Black Sea and Sea of Azov: *Ligophorus acuminatus* Euzet and Suriano, 1977; *Ligophorus cephali* Rubtsova, Balbuena, Sarabeev, Blasco-Costa and Euzet, 2006; *Ligophorus chabaudi* Euzet and Suriano, 1977; *Ligophorus confusus* Euzet and Suriano, 1977; *Ligophorus heteronchus* Euzet and Suriano, 1977; *Ligophorus imitans* Euzet and Suriano, 1977; *Ligophorus macrocolpos* Euzet and Suriano, 1977; *Ligophorus mediterraneus* Sarabeev, Balbuena and Euzet 2005; *Ligophorus minimus* Euzet and Suriano, 1977; *Ligophorus szidati* Euzet and Suriano, 1977; *Ligophorus vanbenedenii* Euzet and Suriano, 1977; *Ligophorus llewellyni* Dmitrieva et al. 2007; *Ligophorus pilengas* Sarabeev and Balbuena, 2004 and *Ligophorus angustus* Euzet and Suriano, 1977. The sample size for each species was 20 individuals for ventral and 20 individuals for dorsal anchors (not necessarily matching specimens of the previous group), except in *L*. *angustus*, where only 4 individuals for ventral and none for dorsal anchors could be studied, and so dorsal anchor was left out of the analysis for this species. In all, 524 anchors were studied of which, in 238 instances, represented ventral and dorsal anchors of the same worm individual.

The present study covers all six grey mullets species reported as host of *Ligophorus* spp. in the Mediterranean, Black Sea and Sea of Azov, including the so-iuy mullet *Liza haematocheila*, which was introduced in the Black Sea and Sea of Azov from the Pacific in the early 1980s [34].

The parasite specimens were collected in the frame of previous studies of our group [22, 27–31] in two marine areas of the Spanish Mediterranean Coast (the Ebro Delta, and Santa Pola Bay), a coastal Mediterranean lagoon (L’Albufera), and the Sea of Azov (Kerch Strait). In addition, part of the specimens of *L*. *llewellyni* and *L*. *pilengas* were collected in the Sea of Japan (Artemovka Delta), i.e., in the host’s native area. (Geographical details of all localities are given in S1 Table). Gills were examined for parasites as per Rodríguez-González et al. [32].

### Geometric morphometrics

#### Morphological data acquisition and landmarks superimposition

Only the anchors were considered for geometric morphometrics techniques because they are not subjected to large variation due to contraction or flattening on fixation [35]. The bars were not studied because they are more difficult to observe flat and more prone to distortion during fixation and mounting. We used photographs and drawings only for ventral and dorsal anchors of partly digested individuals following Rodríguez-González et al. [32]. Any anchor showing apparent deformation, tear or rupture (about 2–3% of the initial sample) was excluded from the study.

To detect outliers in our sample the squared Mahalanobis distance was plotted against the quantiles of the chi-squared distribution [36]. In all, 13 outliers for ventral anchors and 13 outliers for dorsal anchors were identified (see S1 Dataset).

Anchor shape was characterized using landmark-based geometric morphometrics [37]. We digitized 8 landmarks in 2D covering the anchor surface selected and recorded in each anchor using tpsDig version 2.17 [38] representing homologous points (see Figure 1 in Rodríguez-González et al. [32]). Generalized Procrustes analysis in MorphoJ was employed to obtain a matrix of shape coordinates (S1 Dataset) from which all information related to position, scale and orientation were removed [39]. Centroid size, the summed squared distances of each landmark from the centroid of the form was used as a measure of size [40]. The matrix of Generalized Procrustes Analysis coordinates of the ventral and dorsal anchors were subjected to Principal Component Analysis (PCA) based on the covariation matrix. To visualize the variation in shape, we used the first two principal components (PC1 and PC2).

### Quantifying the influence of size on anchor shape

The effects of size on interspecific variation in anchors shape of *Ligophorus* spp. (i.e. interspecific allometry) were tested separately for ventral and dorsal anchors by multivariate regression analyses [34]. We regressed the Procrustes shape coordinates of ventral and dorsal anchors on their log-transformed centroid size (logCS) by means of a multivariate regression through the origin [35, 41]. Then, we mapped the residuals from this regression onto the phylogenetic tree of the parasites. A large difference between the original datasets and the residuals would indicate that evolutionary allometry is an important factor in anchors evolution in *Ligophorus*.

The effect of size on shape was also assessed with phylogenetic independent contrast (PIC) correction [42] in order to avoid incorrect interpretations due to a violation of the assumption of independent sampling [43]. However, no evidence for allometry in any of the PIC-corrected analyses was found significant (*P* > 0.3 in both cases) and, therefore, the effect of evolutionary allometry was not further considered.

### Assessing phylogenetic signal in anchor shape and size

Phylogenetic signal was assessed by mapping a topology of the phylogenetic tree of our 14 species of *Ligophorus* based on a previous published concatenated 28S rDNA and ITS1 phylogeny [27] onto the first two principal component scores of shape and size-corrected shape, and onto logCS representing anchor size. This required an ancestral state reconstruction of the morphometric data for each internal node on the tree using squared change-parsimony assuming a Brownian-motion model of evolution [44].

Phylogenetic signal was tested with MorphoJ [36], where the sum of squared changes of shape along the branches of the tree is minimized over the entire phylogeny. The significance of phylogenetic signal was established by a permutation test in which the topology was held constant and the principal component scores for each taxon were randomly permuted 10,000 times across the tree [45, 46].

The previous analyses provided values of tree length that are inversely related to the strength of the correlation between shape or size and phylogeny [46]. In addition, due to the current controversy on which method is more appropriate to evaluate phylogenetic signal [8], we also used *K*_*mult*_, which is a generalization of Blomberg’s *K* [8, 47]. The main advantage of this approach is that, in addition to informing whether there is a small or large amount of signal present in data, they provide a reference value for departure from the Brownian-motion model of evolution [48]. *K*_*mult*_ = 0 indicates no phylogenetic signal, *K*_*mult*_ = 1 corresponds to phylogenetic signal in the data and that the trait distribution perfectly conforms to the Brownian’s model of trait evolution, values of *K*_*mult*_ < 1 correspond to phenotypic variation that is larger than expected between taxa of the same lineage, and *K*_*mult*_ > 1 indicates stronger similarities among closely related species than expected under the Brownian’s model. The significance of *K*_*mult*_ was evaluated based on comparison of the observed value with those obtained in 999 randomizations [49]. The calculation were performed with function *physignal* in the geomorph package v.3.0.1. [8] in R version 3.2.3 [50].

## Results

### Phylogenetic signal in anchor shape and anchor size

The PCA based on the covariance matrix of landmark data of both ventral and dorsal anchors showed that a large proportion of the variation is contained in relatively few dimensions, with the first two PCs accounting for over a half of the total variance in the sample (Table 1). The first two axes described 68.9% and 52.1% % of the total shape variation (uncorrected for size) and 66.3% and 49.99% of the total shape variation (size-corrected) in ventral and dorsal anchors, respectively (eigenvalues and variance explained by each principal component are given in S2 and S3 Tables). The anchor shapes in our sample were distributed in all shape tangent space for both ventral and dorsal anchors, which are surrounded by distant species from the average shape.

**Table 1:** PCA of variation among the shapes of species mean for ventral and dorsal anchors of *Ligophorus* spp. for original and size-corrected shape.

	Size-uncorrected		Size-corrected	
Anchor	Eigenvalue	Total variance (%)	Eigenvalue	Total variance (%)
Ventral				
PC1	1.23 ·10^−2^	55.7	1.07 ·10^−2^	53.7
PC2	2.90 ·10^−3^	13.1	2.52 ·10^−3^	12.7
Dorsal				
PC1	5.08 ·10^−3^	35.8	4.85 ·10^−3^	33.4
PC2	2.20 ·10^−3^	16.3	2.04 ·10^−3^	16.5

**Fig 1:**
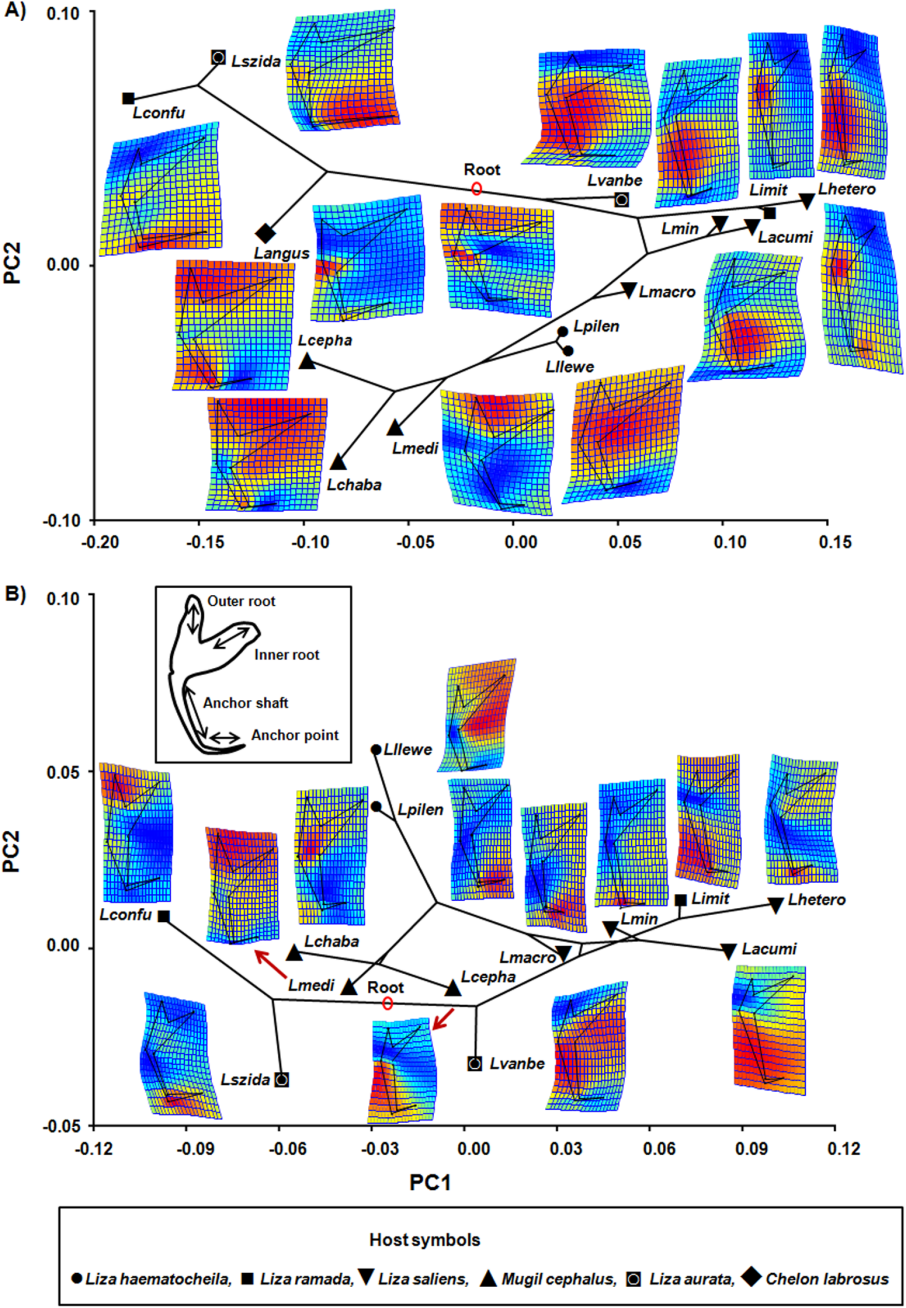
**Projection of phylogeny of the species of *Ligophorus* studied onto the morphospaces of ventral (A) and dorsal anchors (B).** Estimated changes in anchor shapes are shown as Thin-plate-spline deformation grids with color-scaled coded Jacobian expansion factors (red for factors > 1, indicating expansion; strong blue for factors between 0 and 1, indicating contraction) were used. The insert shows the parts of an anchor in *Ligophorus* spp. The ventral anchors of all species included in the analysis are labeled: *Lconfu*: *Ligophorus confusus*, *Lszida*: *Ligophorus szidati*, *Langus*: *Ligophorus angustus*, *Lvanbe*: *Ligophorus vanbenedenii*, *Limit*: *Ligophorus imitans*, *Lhetero*: *Ligophorus heteronchus*, *Lacumi*: *Ligophorus acuminatus*, *Lmin*: *Ligophorus minimus*, *Lmacro*: *Ligophorus macrocolpos*, *Lpilen*: *Ligophorus pilengas*, *Lllewe*: *Ligophorus llewellyni*, *Lmedi*: *Ligophorus mediterraneus*, *Lchaba*: *Ligophorus chabaudi*, *Lcepha*: *Ligophorus cephali*.

The molecular phylogeny of *Ligophorus* spp. projected onto the morphospace defined by the first two PCs of the ventral and dorsal anchor shape is shown in Fig 1. This resulted, respectively, in tree lengths of 0.045 and 0.024, measured in units of squared Procrustes distance along all branches. The deformation grids of each species showing departure from the average anchor shape are also shown.

The projection of the phylogenetic trees onto the morphospaces of ventral and dorsal anchors (Fig 1) showed crossing of branches and some evidence of relatively long branches between related species for ventral and dorsal anchors of species of *Ligophorus*. However, the permutation tests of PC scores revealed significant phylogenetic structure for shape in both ventral and dorsal anchors (*P* < 0.0001 in both cases). Likewise, the K_*mult*_ values were significantly greater than zero (ventral anchors: K_*mult*_ = 0.93, *P* = 0.001; dorsal anchors: K_*mult*_ = 0.5, *P* = 0.011). In fact, in both ventral and dorsal anchors clades occupied specific regions of shape space, which is indicative of phylogenetic structure in the data [44] (Fig 1). In ventral anchors, interspecific variation was caused by the different position of anchors of different clades (Fig 1A). The clade formed by *L*. *confusus*, *L*. *szidati* and *L*. *angustus* was characterized by a long point, short shaft and long inner root (see parts of anchors in insert in Fig 1), the three species occur each on different hosts (*Liza ramada*, *Liza aurata* and *Chelon labrosus*, respectively). A second clade formed by *L*. *cephali*, *L*. *chabaudi* and *L*. *mediterraneus* from *M*. *cephalus*, and by *L*. *pilengas* and *L*. *llewellyni* from *Lz*. *haematocheila* was characterized by large outer roots and short points. Within this clade the anchors of species on *M*. *cephalus* could be distinguished from those occurring on *Lz*. *haematocheila* by the larger outer roots. Two other clades comprising *L*. *imitans* and *L*. *heteronchus*, and *L*. *acuminatus* and *L*. *minimus*, together with *L*. *macrocolpos* exhibited elongated ventral anchors with short points, relatively short inner and outer roots and long shafts. These species are found on *Liza saliens*, except *L*. *imitans*, that occurs on *Lz*. *ramada*. Finally, the shape of anchors of *L*. *vanbenedenii* occurring on *Lz*. *aurata* is intermediate between that of the last five species and that of the *L*. *confusus–L*. *angustus* clade, which is consistent with the phylogenetic position of this species (Fig 1A). In contrast to ventral anchors and although the spatial arrangement of clades in the morphospace was very similar, shape variation in dorsal anchors was more unpredictable as the deformation grids showed quite different patterns at the species level (Fig 1B). As a result, specific shapes could not be clearly associated with particular clades.

**Fig 2:**
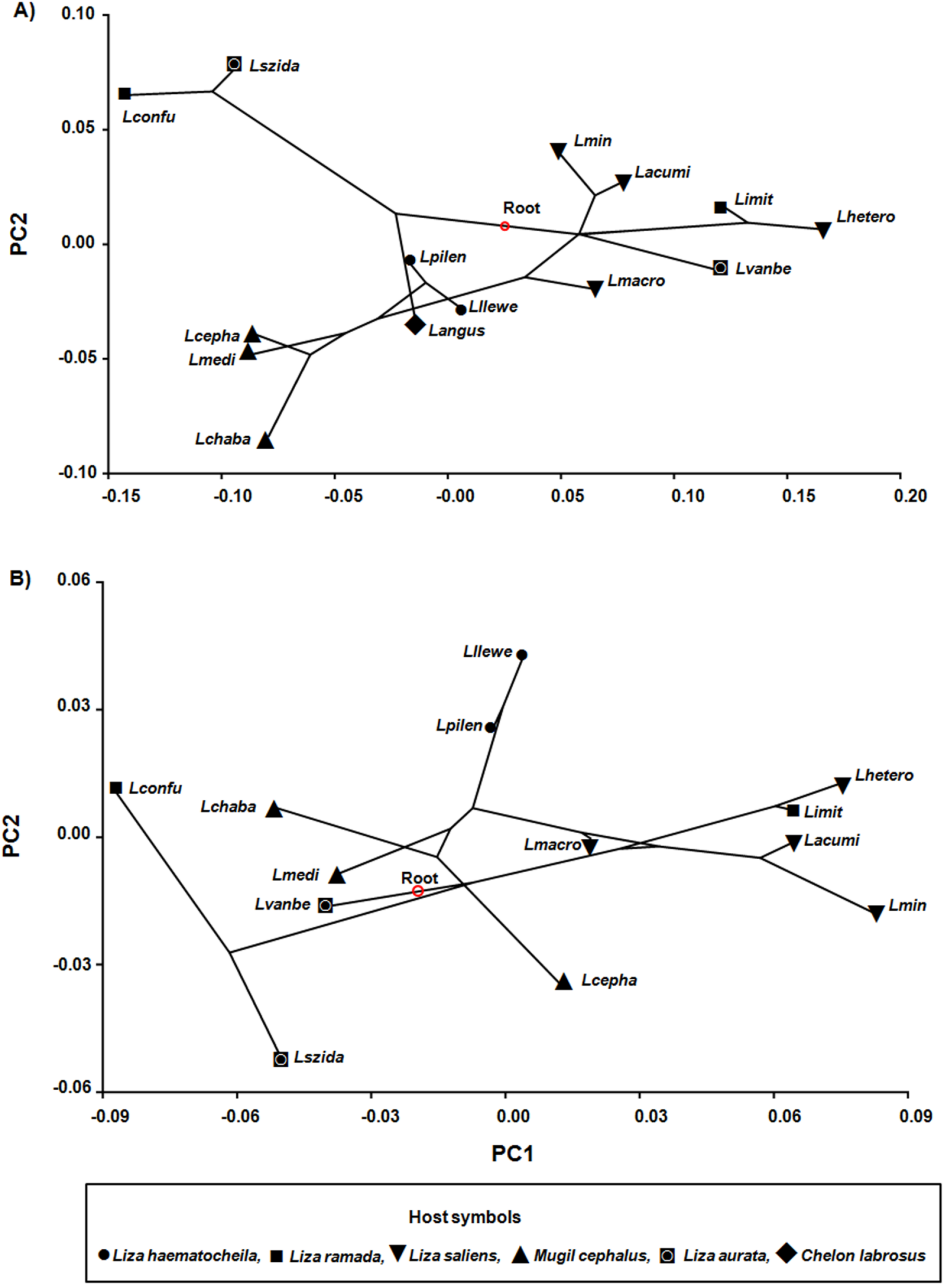
**Projection of phylogeny of 14 species of *Ligophorus* spp. onto the morphospaces corrected for size of ventral (A) and dorsal anchors (B).** Species abbreviations as in Fig 1.

The phylogeny projected onto the first two dimensions of the allometry-free (size-corrected) PCA morphospace of anchor shape yielded tree length of 0.02 for ventral and dorsal anchors, respectively (Fig 2). The highly significant multivariate regression of Procrustes coordinates on logCS (*P* < 0.001) provided evidence for allometric relationships between shape and size in both types of anchors. This relationship accounted for 9.2% and 4.9% of the total shape variation of ventral and dorsal anchors respectively. Again phylogenetic signal was highly significant (*P* < 0.0001 and *P* = 0.0015 respectively). According to the phylogenetic signal with K_*mult*_ (size-corrected), the results were significant (ventral: K_*mult*_ = 0.76, *P* = 0.001; dorsal: K_*mult*_ = 0.77, *P* = 0.002). The scatterplot of ventral anchors (Fig 2A) showed larger branches of *L*. *angustus*, *L*. *macrocolpos*, *L*.*vanbenedenii*, and *L*. *heteronchus* than in the PCA uncorrected for size (Fig 1A). Likewise, for the dorsal anchors (Fig 2B), the branches of *L*. *szidati*, *L*. *confusus*, *L*. *cephali*, *L*. *chabaudi*, *L*. *minimus*, *L*. *llewellyni* and *L*. *heteronchus* were larger than the original PCA (Fig 1B). However, in both cases the position of species in the shape space was similar to the arrangement shown in Fig 1. Therefore allometry had a moderate effect on the overall variation of anchors shape.

**Fig 3:**
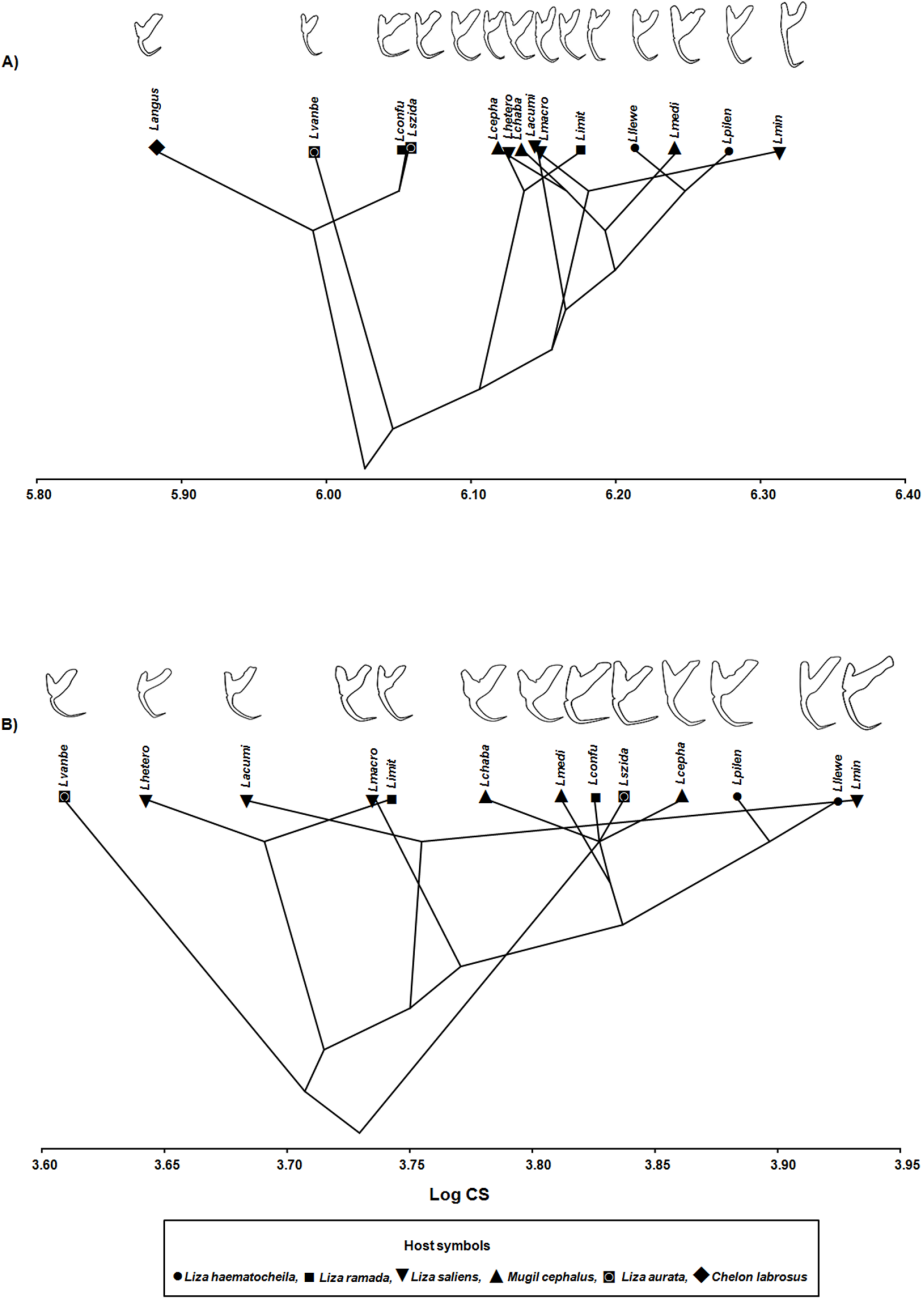
**Projection of phylogenetic tree of 14 *Ligophorus* spp. onto *log* Centroid Size (LogCS) of ventral (A) and dorsal (B) anchors.** Species abbreviations as in Fig 1. The anchors displayed are scaled as per the LogCS scale to convey the gradient in size.

The molecular phylogeny projected onto the gradient in size (logCS) of ventral and dorsal anchors is shown in Fig 3, where the cumulative branch length from the root of the tree is displayed vertically. This mapping resulted in tree lengths of 0.048 and 0.066, for ventral and dorsal anchors respectively, measured in units of logCS distance along all branches. In ventral anchors, *L*. *angustus* showed the larger branches and were separated from all other species, indicating a smaller anchor size than in the other species. Phylogenetic signal tested by random permutation of logCS was statistically significant (*P* < 0.001) in ventral anchors (Fig 3A), but not in dorsal ones (Fig 3B) (*P* = 0.241), whereas Adams [8] K_*mult*_ indicated a significant phylogenetic signal in both anchors (ventral: K_*mult*_ = 1.27, *P* = 0.01; dorsal: K_*mult*_ = 0.6709, *P* = 0.017).

## Discussion

This paper delivers a framework to study the evolution of attachment organs in monogeneans and paves the way for further studies addressing the relationships among morphology, phylogeny and other traits, such as host specificity or niche occupancy. Patterns of morphological change in haptoral anchors were interpreted to reconstruct the dynamics of the evolutionary processes and were visualized as paths from ancestors to descendants through the morphospace of ventral and dorsal anchors.

Given the variety of anchors shapes in *Ligophorus* [29]; it is not surprising that they cover a substantial range of shapes in the tangent space (Figs 1 and 2). The tests performed provided strong evidence for phylogeny playing a major role in determining the shape and, to a lesser degree, the size of the haptoral anchors, which fully agrees with previous work showing a consistent relationship between morphology and phylogeny in *Ligophorus* [18, 22, 30].

Many monogeneans, including the members of *Ligophorus*, are known to be highly host-specific [29], which implies a close interaction with their host. Given that the host can have an influence on genetic and morphological differentiation of monogeneans [51], it has been often hypothesised that haptor morphology reflects adaptations to attachment to the host [24]. This hypothesis can be assessed in this geometric morphometrics framework by comparing the position in the phylomorphospace of distantly related species co-occurring on a given host species. *L*. *confusus* and *L*. *imitans* parasitizing *Lz*. *ramada* represent different clades and their anchors fell far apart in the shape and size morphospaces. Similarly, *L*. *szidati* and *L*. *vanbenedenii* co-occurring on *Lz*. *aurata*, and placed in different clades, differed markedly in shape of the dorsal anchor and size of the dorsal anchor (Figs 1 and 2). Therefore, we found no clear evidence for host-driven homoplasy in the *Ligophorus* spp. studied. However, only these two instances could be analysed and, as discussed below, specific positions in the gills by each species should also be considered.

*Ligophorus* and Mugilidae define an interesting scenario of host parasite associations. Each species of *Ligophorus* predominantly occurs on a single host species and that often co-occurs with one or more congeneric species [30]. In several instances, members of clades that occur on the same host species showed similar anchor forms (*L*. *cephali*–*L*. *mediterraneus* on *M*. *cephalus*, *L*. *llewellyni* and *L*. *pilengas* on *Lz*. *haematocheila*) or similar shapes (*L*. *acuminatus* and *L*. *minimus* on *Lz*. *saliens*). These clades probably resulted from several synxenic speciation events [52].

In addition, sister species occurring on different hosts showed similarities in shape, sometimes also in size, of anchors (compare, for instance, anchor forms of *L*. *imitans* and *L*. *szidati* with those of their respective sister species *L*. *heteronchus* and *L*. *confusus* (Figs 1 and 2). The phylogenetic position of *L*. *imitans*, showing affinities with species found on *Lz*. *saliens*, suggests that its occurrence on *Lz*. *ramada* represents a host-switch. The most ancestral clade formed by *L*. *angustus*, *L*. *confusus* and *L*. *szidati* is also result of host-switch evolutionary events, as each monogenean species of the clade occurs on different mullet hosts. So adaptation to a new host species did not impose dramatic changes in haptoral anchor morphology and the morphological similarities observed point to the occurrence of phylogenetic constrains on anchor form, as proposed for other monogeneans, such as *Lamellodiscus* spp. [51] and *Cichlidogyrus* spp. [53].

Our geometric morphometrics approach also allows identifying paths of morphological evolution. For example, within the *L*. *heteronchus*–*L*. *cephali* clade (corresponding to clade II of Blasco-Costa et al. [30]), the basal species (*L*. *heteronchus* to *L*. *macrocolpos*, predominantly associated to *Lz*. *saliens*, possess narrow anchors with long shafts. This shape would therefore represent the ancestral state relative to the morphologically derived anchors of the *L*. *llewellyni–L*. *cephalis* clade, which includes forms on *Lz*. *haematocheila* and *M*. *cephalus* characterized by larger roots. Roots provide the bases for muscle attachment, so that the force is exerted through muscles and transmitted to the point controlling the anchor grip strength on the gills [27]. Given that *Lz*. *haematocheila* and *M*. *cephalus* represent the largest host species in the present study [54], one can venture the hypothesis that larger roots were evolved for greater grip in order to withstand stronger water currents [22]. In any case, the similarities in anchor morphology of the species occurring on *M*. *cephalus* with those occurring on the Pacific *Lz*. *haematocheila* support the idea that the occurrence of *Ligophorus* in *Mugil* can be explained by a host-switch from the *Liza–Chelon* clade that occurred outside the Mediterranean basin [30].

The evidence for phylogenetic signal was more compelling for the ventral anchors, than for the dorsal ones. This is perhaps not surprising given that dorsal and ventral anchors in *Ligophorus* form two relatively independent evolutionary modules [27]. Empirical evidence from *L*. *cephali* indicates a tighter control of the shape and size in ventral anchors perhaps because they seem to be responsible for firmer attachment [31, 32]. Thus the differences observed could be explained in terms of different functional roles in attachment to the gills [27]. In the present study, the K_*mult*_ corresponding to the shape of dorsal anchors was clearly < 1, which indicates that phenotypic variation is larger than expected between taxa of the same lineage [8]. It has been suggested that a certain degree of homoplasy could account for low K_*mult*_ values of anchor shape in monogeneans [18]. Although the deformation grids do not provide clear evidence for this (Fig 1B), there might still be some hidden homoplasy at the level of within-host microhabitats. Microhabitat was not considered in the present effort because information concerning *Ligophorus* spp. is very scarce [55–57]. Previous work has shown that *L*. *szidati* and *L*. *vanbenedenii* on *Lz*. *aurata*, and *L*. *parvicirrus* on *Lz*. *ramada* differ in their location in the gills [55–57] and, as representatives of different clades, possess distinct morphologies of their attachment organs as discussed above. In addition, Rodríguez-González et al. [32] showed that random effects such as gill section-host individual are important determinants of shape variation in ventral anchors in *L*. *cephali*. So a combination of host species, individual host and microhabitat might contribute to explain the high diversity of dorsal anchor shapes observed (Fig 1B). In any case, if microhabitat information becomes available, it can be readily incorporated into the analyses and future studies of monogeneans can greatly benefit from this approach.

In this study, we have demonstrated that variation of shape and size of the ventral and dorsal anchors in 14 *Ligophorus* spp. is largely determined by common descent and shared evolutionary history, although homoplasy dictated by adaptations to the individual host or to specific gill microhabitats could not be ruled out completely. These two processes may act differently in other monogeneans, but similar analyses of variation in haptoral form as those presented herein can decisively contribute to our understanding of the evolution of attachment organs in monogeneans [18, 20, 53, 58, 59] and other parasites in general. In particular, the adoption of the present approach can help bridge the gap between micro and macroevolutionary processes. Haptoral morphology determines, within one individual host, the specific microhabitats on the gills that, in turn, can influence the specialization and the reproductive isolation among conspecifics through niche segregation [24, 60, 61]. We therefore expect that the present work stimulates further investigations in this area.

## Supporting information

S1 Dataset Raw and procrustes coordinates. (XLSX)

S1 Table Species of *Ligophorus* used in this study collected from five localities: Ebro Delta (40°30′–40°50′N, 0°30′–1°10′E); Santa Pola Bay (38°00′–38°20′N, 0°10′–0°40′W); L’Albufera (39°20′0″N– 0°21′0″W); Kerch Strait, Sea of Azov (45°16′20.8″N–36°31′40.6″E); and Artemovka Delta, Sea of Japan (43°18′30.3″N–132°17′4.8″E). (DOCX)

S2 Table Eigenvalues and associated percent of variance accumulated for each principal component (PC1-12) of shape principal component analyses of ventral anchors corrected and uncorrected for size. (DOCX)

S3 Table Eigenvalues and associated percent of variance accumulated for each principal component (PC1-12) of shape principal component analyses of dorsal anchors corrected and uncorrected for size. (DOCX)
