# Supplementary material for: Evolutionary morphology in shape and size of haptoral anchors in 14 Ligophorus spp. (Monogenea: Dactylogyridae)
Source: PLoS One. 2017 May 24;12(5):e0178367. doi: 10.1371/journal.pone.0178367 (PMC5443544; doi:10.1371/journal.pone.0178367)
Supplement: S1 Table — (DOCX) [file pone.0178367.s002.docx]

| Species of *Ligophorus* | *NV* | *ND* | Host species | Number of fishes | Ebro Delta | Santa Pola | L’Albufera | Kerch Strait | Artemovka Delta |
| --- | --- | --- | --- | --- | --- | --- | --- | --- | --- |
| *Ligophorus acuminatus* Euzet and Suriano, 1977 | 20 | 20 | *Liza saliens* | 7 | X |  |  |  |  |
| *Ligophorus cephali* Rubtsova, Balbuena, Sarabeev, Blasco-Costa and Euzet, 2006 | 20 | 20 | *Mugil cephalus* | 20 |  |  | X |  |  |
| *Ligophorus chabaudi* Euzet and Suriano, 1977 | 20 | 20 | *Mugil cephalus* | 4 | X | X |  |  |  |
| *Ligophorus confusus* Euzet and Suriano, 1977 | 20 | 20 | *Liza ramada* | 5 |  | X |  |  |  |
| *Ligophorus heteronchus* Euzet and Suriano, 1977 | 20 | 20 | *Liza saliens* | 3 | X |  |  |  |  |
| *Ligophorus imitans* Euzet and Suriano, 1977 | 20 | 20 | *Liza ramada* | 3 | X | X |  |  |  |
| *Ligophorus macrocolpos* Euzet and Suriano, 1977 | 20 | 20 | *Liza saliens* | 4 | X | X |  |  |  |
| *Ligophorus mediterraneus* Sarabeev, Balbuena and Euzet, 2005 | 20 | 20 | *Mugil cephalus* | 3 | X | X |  |  |  |
| *Ligophorus minimus* Euzet and Suriano, 1977 | 20 | 20 | *Liza saliens* | 7 | X |  | X |  |  |
| *Ligophorus szidati*, Euzet and Suriano, 1977 | 20 | 20 | *Liza aurata* | 4 |  | X |  |  |  |
| *Ligophorus vanbenedenii* Euzet and Suriano, 1977 | 20 | 20 | *Liza aurata* | 5 |  | X |  |  |  |
| *Ligophorus llewellyni* Dmitrieva, Gerasev and Pron’kina, 2007 | 20 | 20 | *Liza haematocheila* | 4 |  |  |  | X | X |
| *Ligophorus pilengas* Sarabeev and Balbuena, 2004 | 20 | 20 | *Liza haematocheila* | 5 |  |  |  | X | X |
| *Ligophorus angustus* Euzet and Suriano, 1977 | 4 | 0 | *Chelon labrosus* | 3 | X |  |  |  |  |

*NV*, number of ventral anchors; *ND*, number of dorsal anchors.
