# Supplementary material for: Evolutionary morphology in shape and size of haptoral anchors in 14 Ligophorus spp. (Monogenea: Dactylogyridae)
Source: PLoS One. 2017 May 24;12(5):e0178367. doi: 10.1371/journal.pone.0178367 (PMC5443544; doi:10.1371/journal.pone.0178367)
Supplement: S2 Table — (DOCX) [file pone.0178367.s003.docx]

|  |  | **Size-uncorrected** | |  |  | **Size-corrected** | |
| --- | --- | --- | --- | --- | --- | --- | --- |
| **Ventral anchors** | Eigenvalues | % Variance | Cumulative % |  | Eigenvalues | % Variance | Cumulative % |
| **PC 1** | 0.01231297 | 55.75 | 55.75 |  | 0.0106649 | 53.70 | 53.70 |
| **PC 2** | 0.0028931 | 13.18 | 68.93 |  | 0.0025165 | 12.67 | 66.38 |
| **PC 3** | 0.0018328 | 8.65 | 77.58 |  | 0.0018409 | 9.27 | 75.65 |
| **PC 4** | 0.0011447 | 5.22 | 82.81 |  | 0.0011521 | 5.80 | 81.45 |
| **PC 5** | 0.0010554 | 5.04 | 87.85 |  | 0.0010759 | 5.42 | 86.87 |
| **PC 6** | 0.0007714 | 3.47 | 91.32 |  | 0.0007546 | 3.80 | 90.67 |
| **PC 7** | 0.0005305 | 2.80 | 94.13 |  | 0.0006061 | 3.05 | 93.72 |
| **PC 8** | 0.0004351 | 2.10 | 96.24 |  | 0.0004564 | 2.30 | 96.02 |
| **PC 9** | 0.0002761 | 1.37 | 97.61 |  | 0.000281 | 1.42 | 97.43 |
| **PC 10** | 0.0002548 | 1.23 | 98.85 |  | 0.0002624 | 1.32 | 98.75 |
| **PC 11** | 0.0002039 | 0.95 | 99.80 |  | 0.0002051 | 1.03 | 99.79 |
| **PC 12** | 4.296E-05 | 0.19 | 100.00 |  | 4.243E-05 | 0.21 | 100.00 |
