# Supplementary material for: Evolutionary morphology in shape and size of haptoral anchors in 14 Ligophorus spp. (Monogenea: Dactylogyridae)
Source: PLoS One. 2017 May 24;12(5):e0178367. doi: 10.1371/journal.pone.0178367 (PMC5443544; doi:10.1371/journal.pone.0178367)
Supplement: S3 Table — (DOCX) [file pone.0178367.s004.docx]

|  |  | **Size-uncorrected** | |  |  | **Size-corrected** | |
| --- | --- | --- | --- | --- | --- | --- | --- |
| **Dorsal anchors** | Eigenvalues | % Variance | Cumulative % |  | Eigenvalues | % Variance | Cumulative % |
| **PC 1** | 0.01231297 | 35.84 | 35.84 |  | 0.0106649 | 33.49 | 33.49 |
| **PC 2** | 0.0028931 | 16.31 | 52.15 |  | 0.0025165 | 16.50 | 50.00 |
| **PC 3** | 0.0018328 | 12.89 | 65.05 |  | 0.0018409 | 13.54 | 63.54 |
| **PC 4** | 0.0011447 | 11.37 | 76.43 |  | 0.0011521 | 11.93 | 75.48 |
| **PC 5** | 0.0010554 | 8.32 | 84.75 |  | 0.0010759 | 8.53 | 84.02 |
| **PC 6** | 0.0007714 | 4.14 | 88.89 |  | 0.0007546 | 4.25 | 88.27 |
| **PC 7** | 0.0005305 | 3.36 | 92.26 |  | 0.0006061 | 3.56 | 91.84 |
| **PC 8** | 0.0004351 | 2.74 | 95.00 |  | 0.0004564 | 2.90 | 94.74 |
| **PC 9** | 0.0002761 | 2.04 | 97.05 |  | 0.000281 | 2.16 | 96.90 |
| **PC 10** | 0.0002548 | 1.39 | 98.44 |  | 0.0002624 | 1.45 | 98.35 |
| **PC 11** | 0.0002039 | 1.18 | 99.62 |  | 0.0002051 | 1.25 | 99.61 |
| **PC 12** | 4.296E-05 | 0.37 | 100.00 |  | 4.243E-05 | 0.39 | 100.00 |
